# Supplementary material for: Allosteric Modulation of Pathological Ataxin‐3 Aggregation: A Path to Spinocerebellar Ataxia Type‐3 Therapies
Source: Adv Sci (Weinh). 2025 Nov 26;13(11):e02216. doi: 10.1002/advs.202502216 (PMC12931234; doi:10.1002/advs.202502216)
Supplement: Supplementary file 1 — Supporting Information [file ADVS-13-e02216-s001.pdf]

Supporting Information

Allosteric Modulation of Pathological Ataxin-3 Aggregation: A Path to Spinocerebellar Ataxia Type-3 Therapies

Alexandra Silva, Sara Duarte-Silva, Pedro M. Martins, Beatriz Rodrigues, Débora Serrenho, Daniela Vilasboas-Campos, Andreia Teixeira-Castro, Julio Vieyto-Nuñez, Joel Mieres-Perez, Francisco Figueiredo, Martyna Podlasiak, Tatiana-Van-der-Kellen, Joana Fraga, James Noble, Carter Lantz, Niki Sepanj, Daniela Monteiro-Fernandes, Sara Guerreiro, Andreia Neves-Carvalho, Joana Pereira-Sousa, Frank-Gerrit Klärner, Thomas Schrader, Joseph A. Loo, Annalisa Pastore, Elsa Sanchez-Garcia, Gal Bitan, Ana Luísa Carvalho, Patrícia Maciel, Sandra Macedo-Ribeiro*.*

A. Silva and S. Duarte-Silva contributed equally to this work

A. Silva, P. M. Martins, F. Figueiredo, J. Fraga, M. Podlasiak, T. Van-der-Kellen, S. Macedo-Ribeiro
i3S -Institute for Research and Innovation in Health
Institute for Molecular and Cellular Biology (IBMC)
Porto University, 4200-150 Porto, Portugal.
E-mail: sribeiro@i3s.up.pt

S. Duarte-Silva, D. Vilasboas-Campos, A. Teixeira-Castro, D. Monteiro-Fernandes, S. Guerreiro, A. Neves-Carvalho, J. Pereira-Sousa, P. Maciel
Life and Health Sciences Research Institute (ICVS), School of Medicine, University of Minho, ICVS/3B's, PT Government Associate Laboratory, 4710-057 Braga, Portugal.
E-mail:pmaciel@med.uminho.pt

B. Rodrigues, D. Serrenho
Center for Neuroscience and Cell Biology (CNC-UC) & Center for Innovative Biomedicine and Biotechnology (CIBB), University of Coimbra, 3004–504 Coimbra, Portugal.
Institute for Interdisciplinary Research, University of Coimbra, 3004–504 Coimbra, Portugal.

A.L. Carvalho
Center for Neuroscience and Cell Biology (CNC-UC) & Center for Innovative Biomedicine and Biotechnology (CIBB), University of Coimbra, 3004–504 Coimbra, Portugal.
Department of Life Sciences, University of Coimbra, 3000-456 Coimbra, Portugal

J. Vieyto-Nuñez, J. Mieres-Perez, E. Sanchez-Garcia
Department of Biochemical and Chemical Engineering, TU Dortmund University, 44227 Dortmund, Germany.

J. Noble, A. Pastore
King's College London, London WC2R 2LS, United Kingdom.

C. Lantz, J. Loo
Department of Chemistry and Biochemistry University of California, Los Angeles, CA 90095-1569, USA.

N. Sepanj, G. Bitan
Department of Neurology, David Geffen School of Medicine, University of California Los Angeles, Los Angeles, CA 90095-1769, USA.

G. Bitan
Brain Research Institute and Molecular Biology Institute, University of California Los Angeles, Los Angeles, CA 90095-1761, USA.

F. Klärner, T. Schrader
Faculty of Chemistry, University of Duisburg-Essen, 45141 Essen, Germany.

TABLE OF CONTENTS

1. SUPPORTING METHODS 4

1.1. MOLECULAR MODELING STUDIES OF JD:CLR01 COMPLEXES 4
1.2. ATX3 UBIQUITIN HYDROLASE ASSAYS 5
1.3. NUMERICAL METHODS FOR BIOPHYSICAL MODEL OF ATX3 SELF-ASSEMBLY 6
1.4. PHOTO-INDUCED CROSS-LINKING OF UNMODIFIED PROTEINS (PICUP) ASSAY 7
1.5. FILTER RETARDATION ASSAY 7

2. SUPPORTING RESULTS 8

2.1. GAMD SIMULATIONS: EFFECT OF CLR01 ON THE STRUCTURE OF JOSEPHIN DOMAIN (JD) AND PREDICTION OF CLR01 FAVORED BINDING SITES 8
2.2. PICUP ASSAYS AND SEC ANALYSIS OF ATX3 OLIGOMERIZATION 8

3. SUPPORTING FIGURES AND CAPTIONS 10

4. SUPPORTING TABLES 22

5. REFERENCES 31

1. SUPPORTING METHODS

1.1.Molecular modeling studies of JD:CLR01 Complexes

Computational details

The initial coordinates for the GaMD simulations [1] of the JD in the absence and presence of CLR01 molecules were obtained from the first model of the NMR structures with PDB ID: 1YZB [2] and 2AGA [3] (**Figure S2**). The CHARMM-GUI server [4] was employed for the preparation of all systems, generation of their topologies for the CHARMM36m force field [5] and the inputs for AMBER. [6] The protonation states for the titrable protein residues were set at pH 7. The systems were solvated in a rectangular box of TIP3P [7, 8] water molecules with a minimum distance of 15 Å between the protein and the edge of the box. Counterions of NaCl were added for net charge neutralization. The same protocol was applied to study the JD in the presence of CLR01. The JD has in total six lysine and eight arginine residues. In the 1YZB model, two lysine residues (K125, K166) and three arginine residues (R47, R110, and R124) were sterically inaccessible for complex formation with CLR01. In the 2AGA model, two arginine residues (R110 and R124) and one lysine residue (K125) were inaccessible. CLR01 molecules were placed around the remaining lysine and arginine residues, resulting in 1:9 and 1:11 (1YZB or 2AGA): CLR01 complexes, respectively (**Figure S2, right**). Additionally, we prepared a system comprising the JD open model and a single CLR01 molecule positioned at K128. The complexes were simulated following an analogous procedure as for the JD models alone. For CLR01, CHARMM general force field (CGenFF) parameters, [9] tested and used in previous studies, [10] were employed. The particle mesh Ewald (PME) method was used for long-range electrostatic interactions [11] and a cutoff of 12Å for the vdW contributions as implemented in CHARMM-GUI. Each system was subjected to 5000 minimization steps, followed by NVT of 125 ps applying harmonic positional restraints and NPT simulation of 250 ps at 303.15 K and 1 bar of external pressure using Berendsen barostat. Then, 2.4 ns of classical MD were carried out, followed by 8-12 ns of GaMD equilibration until convergence of k0D and k0P to 1. Unrestrained GaMD productions were carried out (3 replicas of 200 ns each) for all the setups using AMBER 20. [12] In the case of the additional system of the JD open in complex with a single CLR01 molecule positioned at K128, the replicas were run for 500 ns of simulation time. The simulations of the JD open structure without the tweezer were extended accordingly. For the analyses of the trajectories and visualization we employed Visual Molecular Dynamics VMD [13] and PyMOL [14] software.

Gibbs Energy Calculations

The CL-FEP approach [15] was employed to investigate the binding of the JD to CLR01. The sampling was performed with independent MD simulations on subsystems that included the ligand (CLR01), the host protein (JD), the complex (JD+CLR01), and the bulk solvent (water box). A harmonic wall potential of 50 kcal/mol between the center of mass (COM) of CLR01 and 2 Å distance of the COM of its binding site in the host was applied. An RMSD restraint of 100 kcal/mol was imposed in the simulation setups of the complex and host. Every subsystem was solvated with water molecules using the TIP3P model. [7] The PME method [11] was employed to account for long-range electrostatic interactions and Na^+ and Cl^- ions were added for neutralization. The initial minimization simulation was carried out for 10,000 steps, followed by NVT equilibration of 75 000 steps. Subsequently, NPT simulations of 250,000 steps and 2fs timestep using harmonic constraints were performed. Nine individual setups (complex, host, ligand, solvent) corresponding to the nine sterically accessible Lys/Arg were generated for the 1YZB model. We carried out three production replicas of 100 ns each for all setups using NAMD. [16] The free energy estimation over the 300 ns of sampling was obtained using the second-order cumulant estimator. The analysis was performed with 15 checkpoints and replicated 30 times. The average free energy values among converged checkpoints with further subtraction of the correction factor provided the final estimation of free energy changes. [15]

1.2. Atx3 ubiquitin hydrolase assays

Atx3 13Q and Atx3 77Q enzymatic activity was assessed by monitoring the release of fluorescent 7-amino-4-methylcoumarin (AMC) from the substrate ubiquitin-AMC (Ub-AMC, UbiQ Bio BV). The enzymatic assays were performed in a reaction buffer of 50 mM HEPES pH 7.5, 0.5 mM EDTA pH 8.0, 0.1 mg/mL bovine serum albumin (BSA), 5% (v/v) glycerol, and 10 mM DTT at 37 ºC using 384-well microplates (black, flat bottom, polystyrene, CORNING®, REF 3573) in a total volume of 50 µL per well. Reactions were initiated by the addition of Ub-AMC to a final concentration between 0.09375 to 1 µM and enzyme concentration 1 µM, both in the presence and absence of 10 µM molecular tweezer CLR01. Prior to substrate addition, Atx3 13Q and Atx3 77Q were pre-incubated with CLR01 for 10 min at 37 ºC. The reaction mixture was covered with 30 µL of liquid paraffin to avoid evaporation. AMC fluorescence was monitored at 460 nm (390 nm excitation) using a CLARIOstar Multi-detection microplate reader (BMG LABTECH).

1.3.Numerical Methods for Biophysical Model of Atx3 Self-Assembly

DLS data measured during Atx3 13Q are well described by a nucleation-and-growth model of phase separation. [17] The main simplifying hypothesis adopted during the derivation of this model are: (i) primary nucleation is described by a second-order rate equation on supersaturation, (ii) growth and secondary nucleation are autocatalytic processes whose rates increase with the mass concentration of particles and are first-order functions of supersaturation, (iii) no significant occurrence of breakage/fragmentation/ coalescence events; (iv) the volume of the supersaturated solution remains approximately constant; (v) no surface tension effects are considered. [18] Under these conditions, the time evolutions of mass $M(t)$ and mean size $N(t)$ of new particles formed by NAG are given by the equations: [17, 18]

$$\alpha(t) = 1 - \frac{1}{k_b[\exp(k_\alpha \Delta\mu_0 t) - 1] + 1} \quad \text{(Equation S1a)}$$

$$\frac{N_1}{N(t)} = \frac{k_b}{1-k_b}\left[\frac{\ln\left(1-\alpha(t)\right) + k_\alpha \Delta\mu_0 t}{\alpha(t)(1-k_b)}\left(1 - \frac{k_2}{k_\alpha}\frac{N_1}{N_2}\right) - \left(1 - \frac{k_2}{k_\alpha k_b}\frac{N_1}{N_2}\right)\right] \quad \text{(Equation S1b)}$$

where $\alpha(t) = M(t)/M_\infty$ is the reaction conversion and M_∞ is the total mass; N_1 and N_2 are the sizes of primary and secondary nucleation particles, respectively; $\Delta\mu_0$ is the initial supersaturation approximated as $\Delta\mu_0 \approx (c_0 - c_\infty)/c_\infty$, with c_0 and c_∞ representing the inital protein concentration and the protein solubility, repectively; and k_α and k_b are functions of the rate constants characterizing the microscopic steps of primary nucleation (k_n), surface growth (k_+) and secondary nucleation (k_2):

$$k_\alpha = (k_+ + k_2) \quad \text{(Equation S2a)}$$

$$k_b = \frac{k_n}{k_+ + k_2} \quad \text{(Equation S2b)}$$

The scattering intensities represented by vertical lines in Figure 2 and Figure S5 are computed assuming the equivalence between $N(t)/N_1$ and $[R(t)/R_1]^3$ ratios, where R refers to hydrodynamic radii. Each particle is modelled as a Mie scatterer, whose scattering intensity is calculated using the MATLAB code *MatScat.* [19]. A good agreement between the NAG model and the DLS data in the absence of CLR01 is obtained by adopting a simplified fitting procedure that assumes $k_2/k_\alpha \approx 1$ (and $k_2 \approx 0$) when the increase in scattering intensity takes place at nearly invariant $R(t)$.Initial estimates of $k_n\Delta\mu_0$, $k_+\Delta\mu_0$ and $k_2\Delta\mu_0$ were obtained from solving Eq. S1 and S2 using the half-life coordinates ($\alpha(t_{50}) = 0.5$) measured during thioflavin-T (ThT) fluorescence experiments. The final values of $k_n\Delta\mu_0$, $k_+\Delta\mu_0$ and $k_2\Delta\mu_0$ were fitted to the measured variations of hydrodynamic radii and scattering intensities of amyloid fibrils over time the function *lsqcurvefit* of MATLAB R2023a (Mathworks, Natick,

MA). While the results in **Figure 2** and **Figure S7a,b** support the validity of our theoretical analysis, the size distributions obtained for long aggregation times exhibit features that the NAG model does not predict. One of these features is the formation of Atx3 77Q aggregates with hydrodynamic radii R_H in the micrometre range (**Figure 2c**). To account for this population of large species, an additional step of fibril agglomeration (that does not increase ThT fluorescence) has to be considered only for the Atx3 77Q variant. Another interesting feature is the population of very small oligomers (R_H=10 nm) observed after 72h incubation for both variants in the presence of CLR01 (**Figure S7a, b**). This result suggests that CLR01 induces additional secondary pathways such as fibril fragmentation or secondary nucleation by which very small oligomers are generated.

1.4.Photo-Induced Cross-linking of Unmodified Proteins (PICUP) assay

PICUP reactions were performed according to previously described protocols, with minor modifications. [20] Briefly, for each reaction, the purified proteins and CLR01 solutions were mixed diluted to a final concentration of 10 µM in the assay buffer (100 mM sodium phosphate, pH 7.4). For each reaction, the protein and CLR01 solutions were mixed to the final concentrations in 18 µL PCR tubes, followed by the addition of 1 µL Ru(Bpy) and 1 µL APS. The final concentration of protein was 10 µM, the CLR01 concentrations were 1, 3, 10, 30 or 100 µM, the concentration of Ru(Bpy) and APS were 40 µM and 800 µM, respectively. The reaction started with irradiation for 1 s, and quenching immediately with 1 µL DTT. Samples were fractionated by SDS-PAGE using Tris-Acetate. Non-cross-linked proteins were used as a control. Protein bands were visualized by silver staining (Invitrogen). Gels were scanned and the abundance of oligomeric species in each lane was calculated by densitometry analysis relative to the entire lane using Image J. The data represents the average of 3 replicates.

1.5.Filter Retardation Assay

At the endpoint of the aggregation assays, monitored by ThT fluorescence, the samples were analyzed using a filter retardation assay according to a previously described protocol, using a mouse anti-Atx3 antibody (1:10000, Millipore, 1H9, Cat # MAB5360, Lot #3700911). [21] Slot-blot signals were visualized using X-ray film with an exposure time of **45 seconds**.

2. SUPPORTING RESULTS

2.1. GaMD Simulations: Effect of CLR01 on the structure of Josephin Domain (JD) and prediction of CLR01 favored binding sites

We performed Gaussian accelerated molecular dynamics (GaMD) simulations to explore the inhibition mechanism of Atx3 aggregation by CLR01. To investigate the favored binding sites of CLR01 in the JD, we analyzed the non-covalent contacts of the tweezer molecule with the amino acids in the JD over the GaMD. The simulations for open conformation in the presence of CLR01 showed several residues with a large population of contacts (**Table S1**). The estimated binding free energy changes for the complexes between CLR01 and selected residues of JD on the open conformation revealed seven sites in which binding of CLR01 to exposed lysine and arginine residues on the JD is favored (**Figure S4c**). Among them, R182, K85, R101, K117, R59, and R103 were thermodynamically favorable according to Gibbs free energy calculations to interact with CLR01, but no chemical-shift perturbations were observed in the NMR data. Previous data has shown that, although arginine residues may be favored electrostatically, lysine residues are sterically more accessible and have consistently shown to have a higher affinity for CLR01,[22] in full agreement with our simulations for JD (**Figure S4b**). In fact, the smaller and less bulky side chain of lysine can thread more easily than arginine into the tweezer's cavity without inducing structural distortions of the torus-shaped arrangement of alternating benzene and norbornadiene rings. [23] Concerning the residues with thermodynamically favored CLR01 interactions, K128 emerges as the preferred CLR01 binding site and has the second highest SASA value for lysine in JD, after K8 (**Figure S4a**). K8 and R182, located at the N- and C-terminus, respectively, were involved in hydrogen bonds with E7 (12.9%) and E10 (14.8%) for the former and with Q129 (13.9%) and D145 (15.0%) for the latter, potentially hindering stable CLR01 binding. For K85, which exhibited a similar SASA value to K128, the occupancy of the hydrogen bond with E90 was 29.1%. The simulations for open conformation in the presence of CLR01 at K128 showed several residues with a large population of contacts close to the termini of the JD, including hydrophobic cluster (L178, M180, I181) that may further stabilize complex formation at this site.

2.2. PICUP Assays and SEC analysis of Atx3 oligomerization

To assess the effect of CLR01 on the initial stages of Atx3 assembly, we used the Photo-Induced Cross-linking of Unmodified Proteins (PICUP) assay. [20] Cross-linked dimers of the

JD, and a range of Atx3 13Q and Atx3 77Q oligomers, including putative dimers, trimers, and tetramers, could be visualized in the PICUP assay (**Figure S6b**). CLR01 weakly decreased the relative abundance of oligomeric species for both Atx3 variants and for the JD. The IC_{50} values were between 170 to 200 μM), corresponding to a 17- to 20-fold molar ratio of the molecular tweezer (**Figure S6b**). The effect of CLR01 on the overall decrease in Atx3 oligomerization was further corroborated by the analysis of the time-dependent distribution of monomer and high molecular weight (HMW) soluble species by size-exclusion chromatography (SEC). CLR01 caused a delay in the conversion of monomers to HMW species for both Atx3 variants (**Figure S6c**). This delay was analyzed quantitatively by comparing the relative peak areas of HMW oligomers and Atx3 monomers. In the presence of CLR01, at the end-point of the aggregation assay (140h), ~50% Atx3 13Q remained in its monomeric form, whereas in the control condition, less than 20% of the monomeric species remained after 90h at 37 °C. Similarly, most Atx3 77Q monomers were converted to HMW species after 72 h, while monomers were still detectable even after 90 h of incubation in the presence of CLR01. This effect was not observed by the incubation with the control molecule, CLR03.

3. SUPPORTING FIGURES AND CAPTIONS

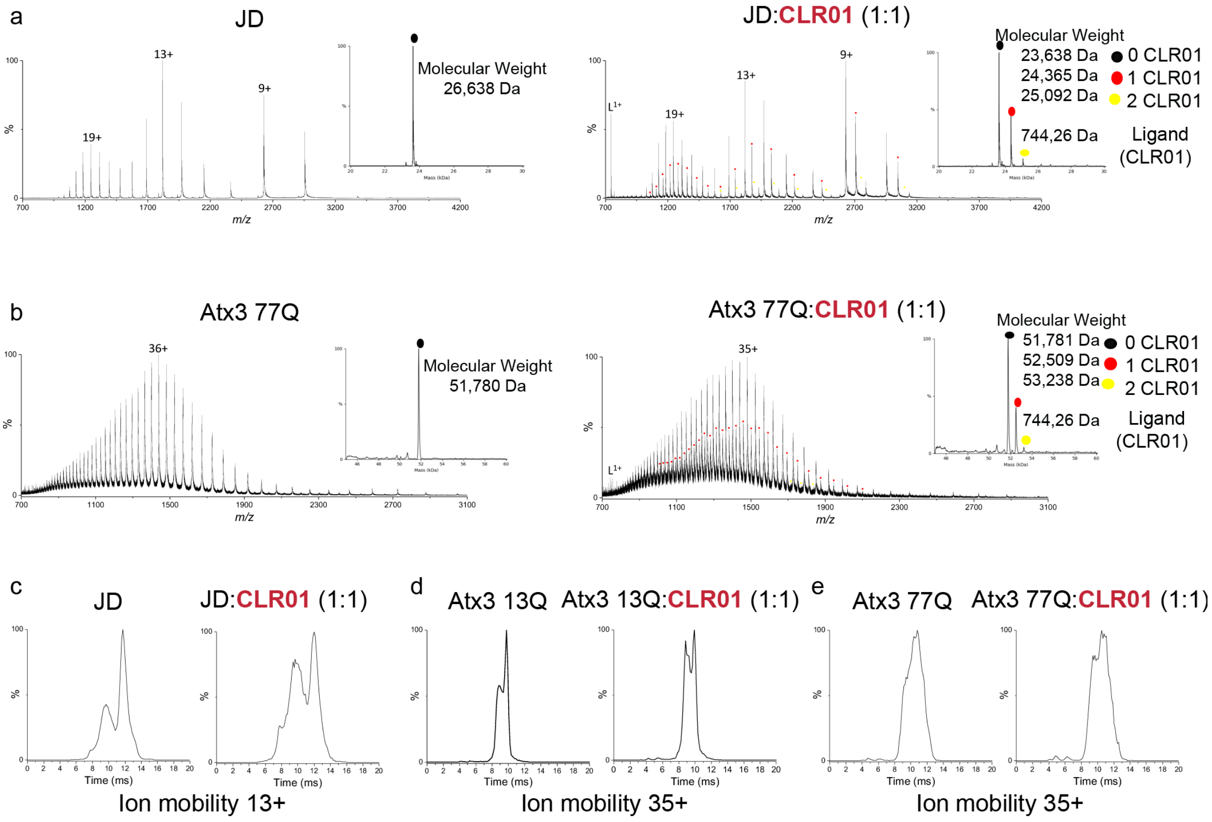

Figure S1 – CLR01 forms a complex with the isolated JD and with full-length Atx3. **a)** ESI mass spectra of JD alone (left) and in complex with CLR01 (right). **b)** ESI mass spectra of Atx3 77Q alone (left) and in complex with CLR01 (right). The insets in a) and b) show the deconvoluted spectra and highlight the two CLR01 binding events. **c)** IM-MS of the 13+ charge state of JD in the absence of presence of CLR01. **d)** IM-MS of the 35+ charge state of Atx3 13Q in the absence of presence of CLR01. **e)** IM-MS of the 35+ charge state of Atx3 77Q in the absence of presence of CLR01.

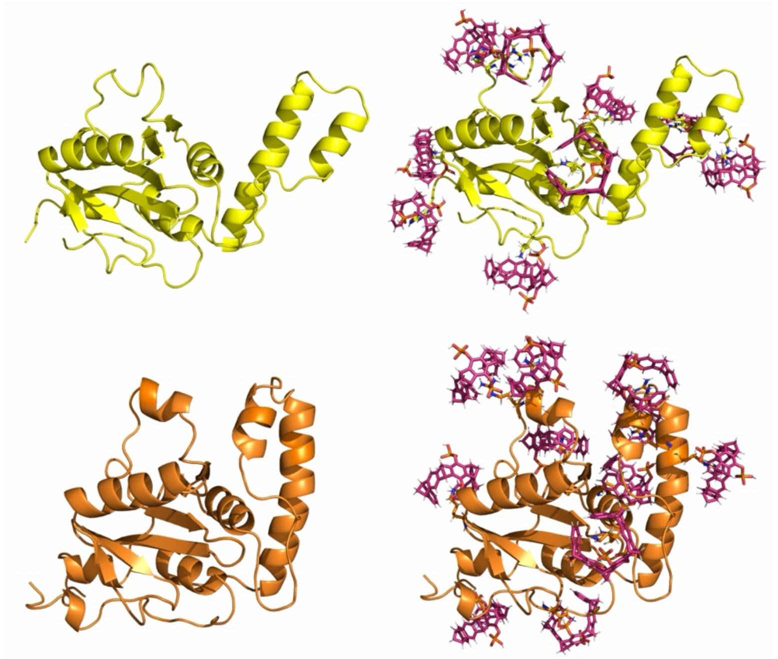

Figure S2 - Initial structures of the JD domain used for the simulations: 1YZB model (open, in yellow) and 2AGA model (closed, in orange) in the absence (left) and presence (right) of CLR01 molecules. Lysine and arginine residues as well as CLR01 are shown in licorice representation.

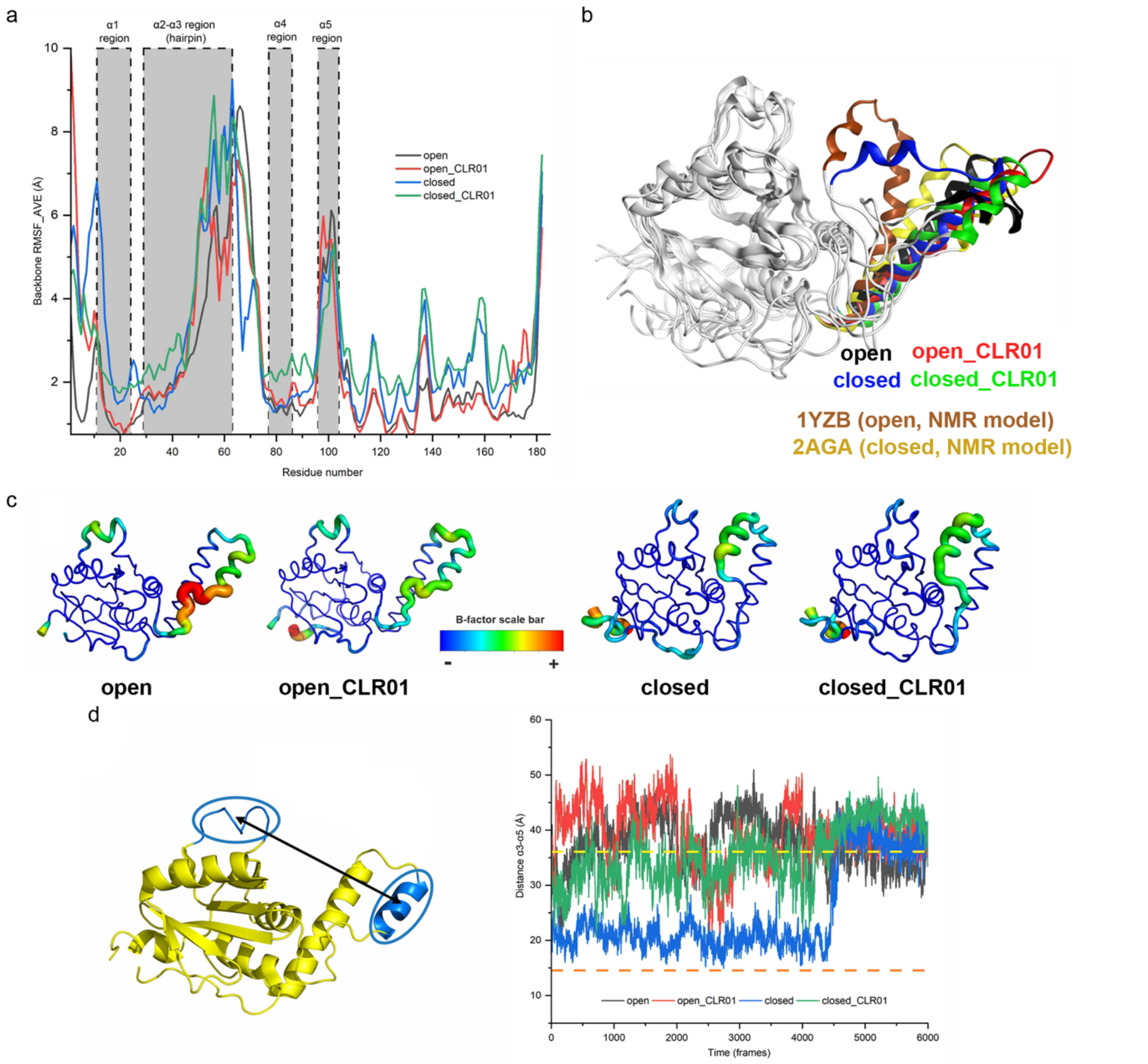

Figure S3 - Conformational variability of JD throughout 600 ns of GaMD simulations in absence or presence of CLR01 molecules. **a)** Root Mean Square fluctuation (RMSF) plot. Each point represents the mean fluctuation per residue calculated over the complete GaMD trajectory. The helixes α1, α2, α3, α4 and α5 are highlighted in grey and dashed boxes. The most dynamic region is the hairpin loop (α2 and α3, S29-Q63), which is responsible for the large-scale conformational change observed in the simulations ($RMSF_{hairpin} > 7$Å. **b)** Superposition of representative structures of the most populated cluster for each simulated system (JD open, JD open_CLR01, JD closed, and JD closed_CLR01). The NMR models 1YZB and 2AGA were included as reference for JD's open and closed states, respectively. **c)** The average backbone B-factor profiles as function of RMSF were calculated from the GaMD trajectories of JD with or without CLR01. The highest B-factor (highest flexibility) for each structure is coloured in red and the lowest in dark blue. The thickness of the protein backbone is also proportional to the B-factors. **d)** Time evolution of the distance values (left panel) between the centre of mass of the α3 and α5 regions of the JD (right panel) in the absence or presence of CLR01. The reference distance values, taken from the NMR models, are represented as two horizontal dash lines for the open (yellow) and the closed (orange) states of the JD. The analysis reveals the inherent flexibility of the JD, exhibiting a coexistence of both states, whereas the JD open_CLR01 and JD closed_CLR01 systems undergo partial or complete transitions to a half-open conformation upon the addition of CLR01. The closed system, in the absence of CLR01 (closed, blue graph), transitioned to the open configuration in one of

the GaMD replicas, in agreement with previous reports of the open form as the favored state of the JD. [24]

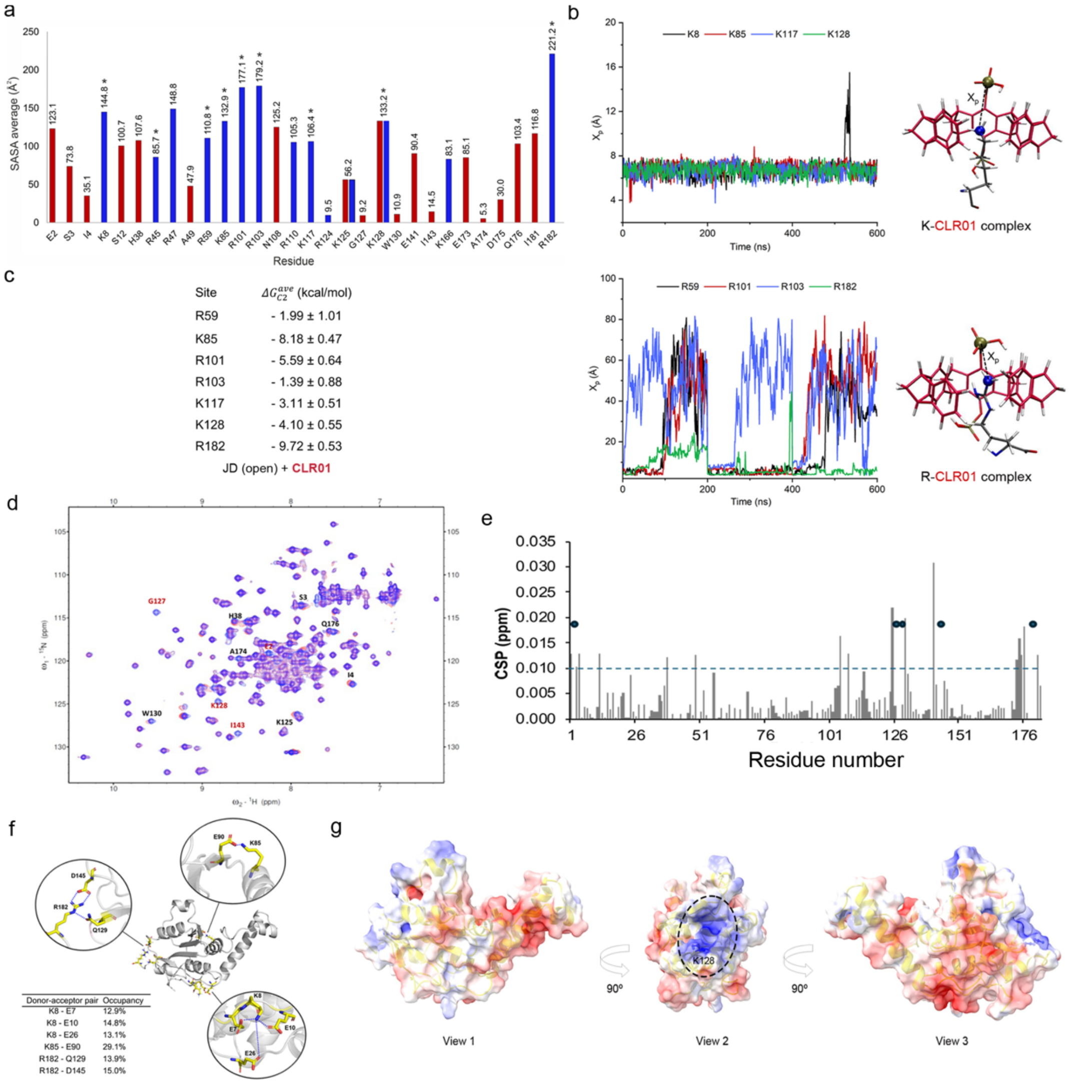

Figure S4 – Computational and experimental analysis of CLR01 interaction with JD. **a)** SASA analysis of all JD (open conformation) R/K residues (blue); residues for which chemical shifts were observed in NMR (panel d) are shown in red. The values on top of the bars show the average SASA for each residue computed over the three GaMD trajectories of JD; the star symbol highlights the R/K residues simulated in the complex with CLR01. **b)** Plot of the X_p geometric parameter corresponding to the distance between the closest nitrogen atom of K/R residues side chain and the phosphorus atom in CLR01. **c)** Binding Gibbs energies (calculated using CL-FEP with the second order cumulant (C2) estimator) for the R/K residues in complex with CLR01 for which binding was predicted to be thermodynamically favourable. **d)** Overlay of SOFAST-HMQC spectrum of JD alone (100 uM; blue) and upon addition of a twofold molar excess of CLR01 (red), recorded at 21°C on a 800 MHz spectrometer. Residue numbers identify resonances with the largest CSPs. **e)** Histogram with the chemical shift perturbation (in ppm) as a function of the amino acid sequence of the JD domain. The values were obtained by weight-averaging the chemical shift variations in the ^1H and ^{15}N dimensions according to the well-established formula $\Delta\delta=(1/2*(\Delta\delta H^2+(0.2\Delta\delta N)^2))^{1/2}$. An arbitrary cutoff at 0.010 ppm (indicated with a dashed line parallel to the x-axis). Residues that disappear are marked with black dots. **f)** Hydrogen bond interactions of K8, K85 and R182, illustrating their most frequent interactions over the simulations. **g)** Electrostatic potential surface of a representative structure from the most populated cluster of JD (open conformation) generated with

APBS. [25] In view 2, the area with the highest positive charge density, localized around residue K128 (in balls and sticks), is highlighted.

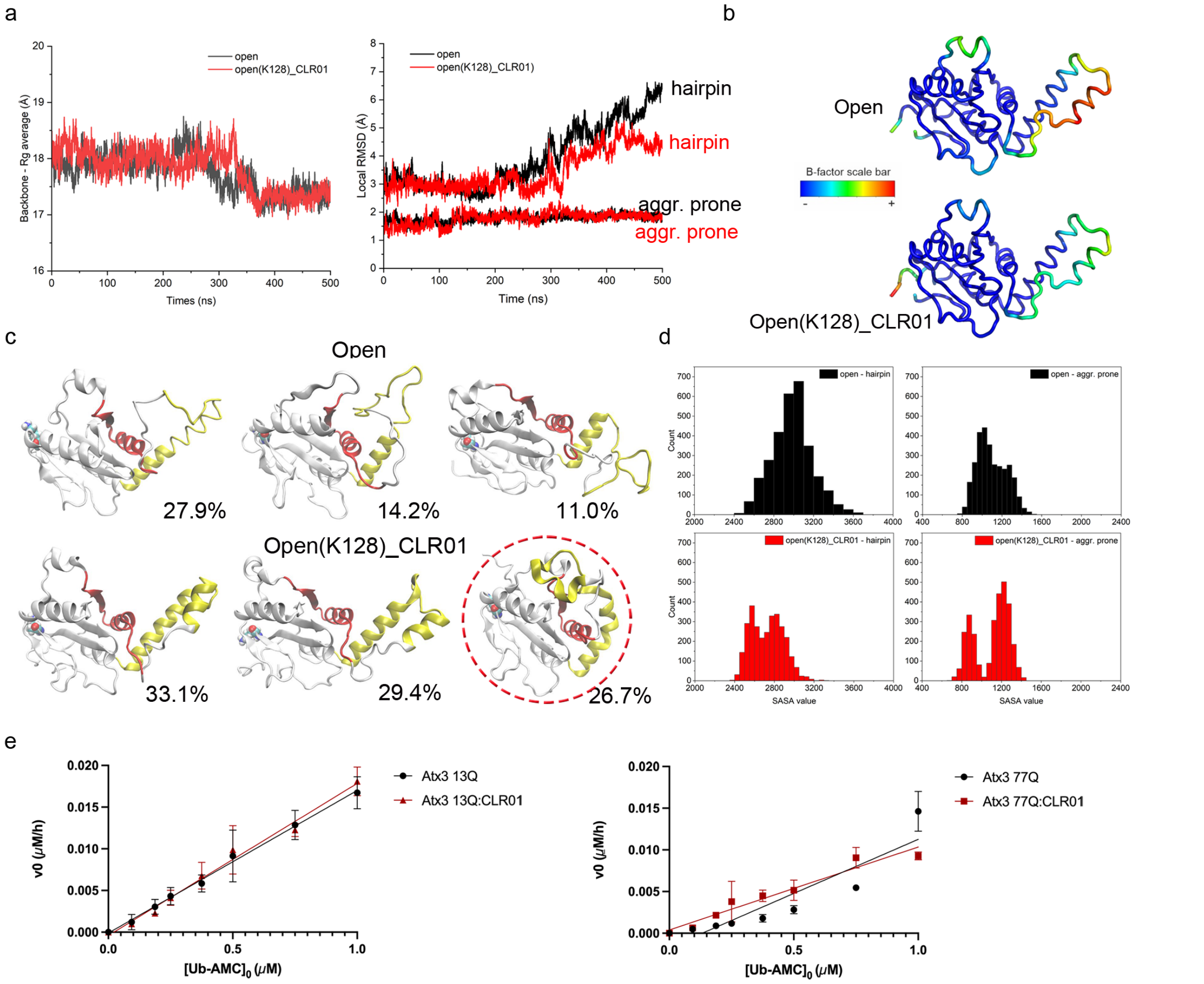

Figure S5 – CLR01 binding to K128 induces an allosteric effect on the JD helical hairpin without affecting Atx3 ubiquitin hydrolase activity. a) Radius of gyration (Rg) of the protein backbone and root-mean-square deviation (RMSD) focused on the helical hairpin and aggregation-prone region. Both analyses represent average values from three GaMD production replicas of 500 ns each and were obtained from simulations of the JD alone or in complex with CLR01 bound to K128. **b)** Average backbone B-factor profiles as a function of root-mean-square fluctuation (RMSF) were calculated from the GaMD trajectories (3x500 ns) for JD alone or in complex with CLR01. The highest B-factors (indicating the largest flexibility) are colored red, while the lowest are dark blue. **c)** Clustering analysis of the last 100 ns from each GaMD replica (3x100 ns) for the JD alone or in complex with CLR01. The centroids of the three most populated clusters of structures (with corresponding population percentages) reveal the high flexibility of the helical hairpin motif. In the JD:CLR01 complex, a state (dashed circle) was identified where the hairpin moves upon the aggregation-prone region, corresponding to a population of 26.7%. **d)** Histograms of solvent-accessible surface area (SASA) values focused on the hairpin and aggregation-prone region over the last 100 ns from each GaMD replica (3x100 ns) for JD or JD:CLR01. The labels on the histogram peaks represent the values with the highest frequency in each case. **e)** Effect of CLR01 (1:10 molar ratio) on the initial rates (v0) of Ub-AMC hydrolysis by Atx3 13Q and Atx3 77Q. Symbols correspond to the initial rates of Ub-AMC hydrolysis measured with (red) or without (black) of 10 µM molecular tweezer CLR01. Error bars correspond to the standard deviations of triplicate measurements. Lines: linear fits to estimate Vmax/Km ratios for Atx3 13Q (0.01711 ±

0.0008936 (without CLR01) and 0.01825 ± 0.0008784 (with CLR01)), and for Atx3 77Q (0.01297 ± 0.001332 (without CLR01) and 0.009949 ± 0.0007778 (with CLR01)). Ub-AMC concentrations were maintained below 1 μM, in accordance with the manufacturer's recommendations.

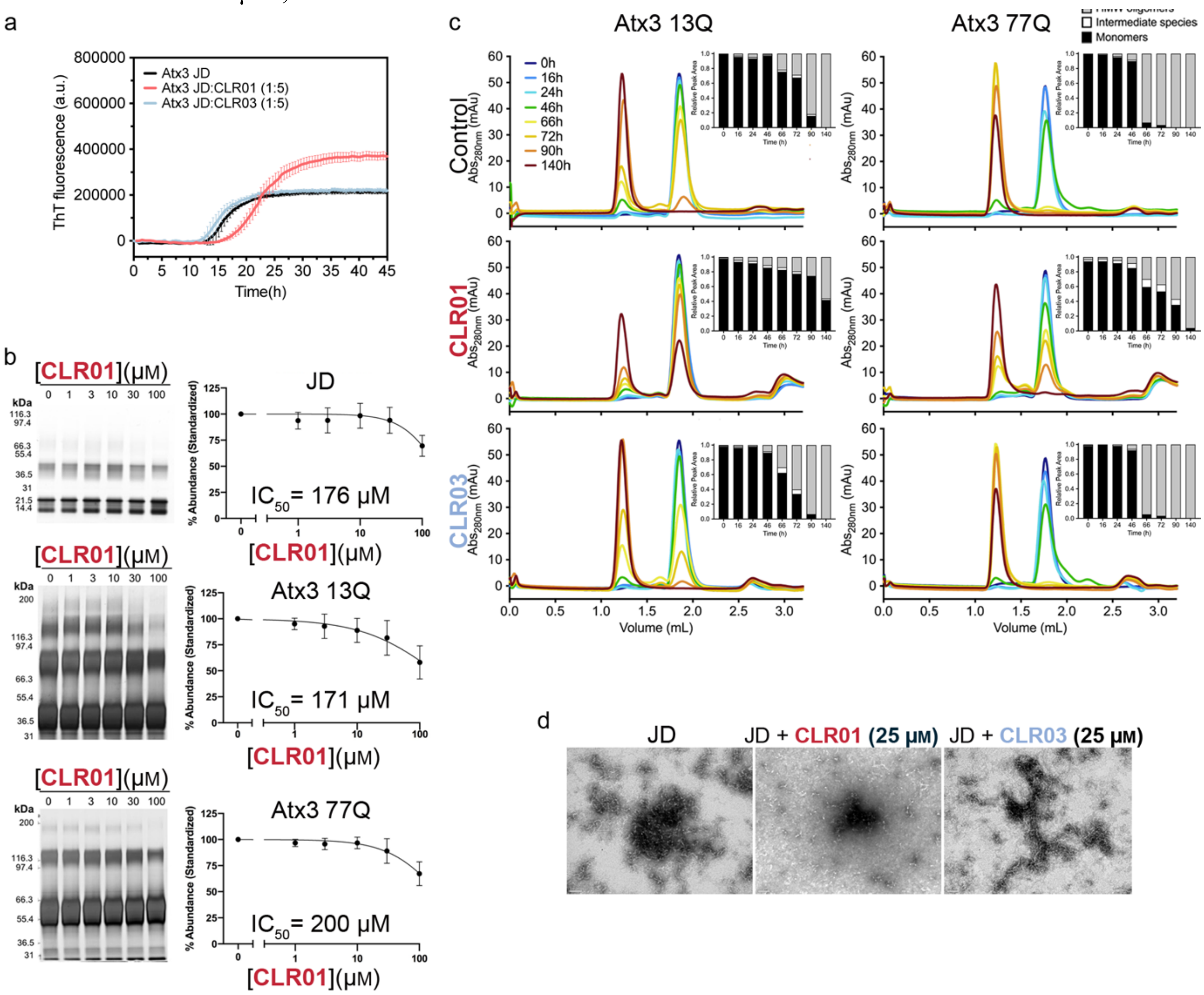

Figure S6 - CLR01 delays the self-assembly of JD and full-length Atx3. a) ThT measurement of JD (5 μM) amyloid self-assembly kinetics in the absence and presence of 5-fold molar excess of CLR01 or CLR03. Error bars correspond to SD of 3 replicates. **b**) Photo-induced cross-linking of unmodified proteins (PICUP) assay of 10 μM JD, Atx3 13Q, or Atx3 77Q in the presence of 0, 1, 3, 10, 30 or 100 μM of CLR01, followed by SDS-PAGE analysis, to determine CLR01 half-maximal inhibitory concentration (IC_{50}) of Atx3 isoforms oligomerization. Error bars correspond to SD of 3 replicates. **c**) Time-course distribution of soluble monomeric and oligomeric species of 5 μM Atx3 13Q or Atx3 77Q in the absence or presence of 25 μM CLR01 or CLR03 monitored by analytical SEC. Relative peak area (RPA) graphical representation of each time point **is presented next to the corresponding SEC profile. d**) Analysis of JD (5 μM) fibrillar structures in the presence of CLR01 (25 μM) or CLR03 (25 μM) at the endpoint of the aggregation assay (t=60h), monitored by negative staining TEM. Scale bars correspond to 100 nm.

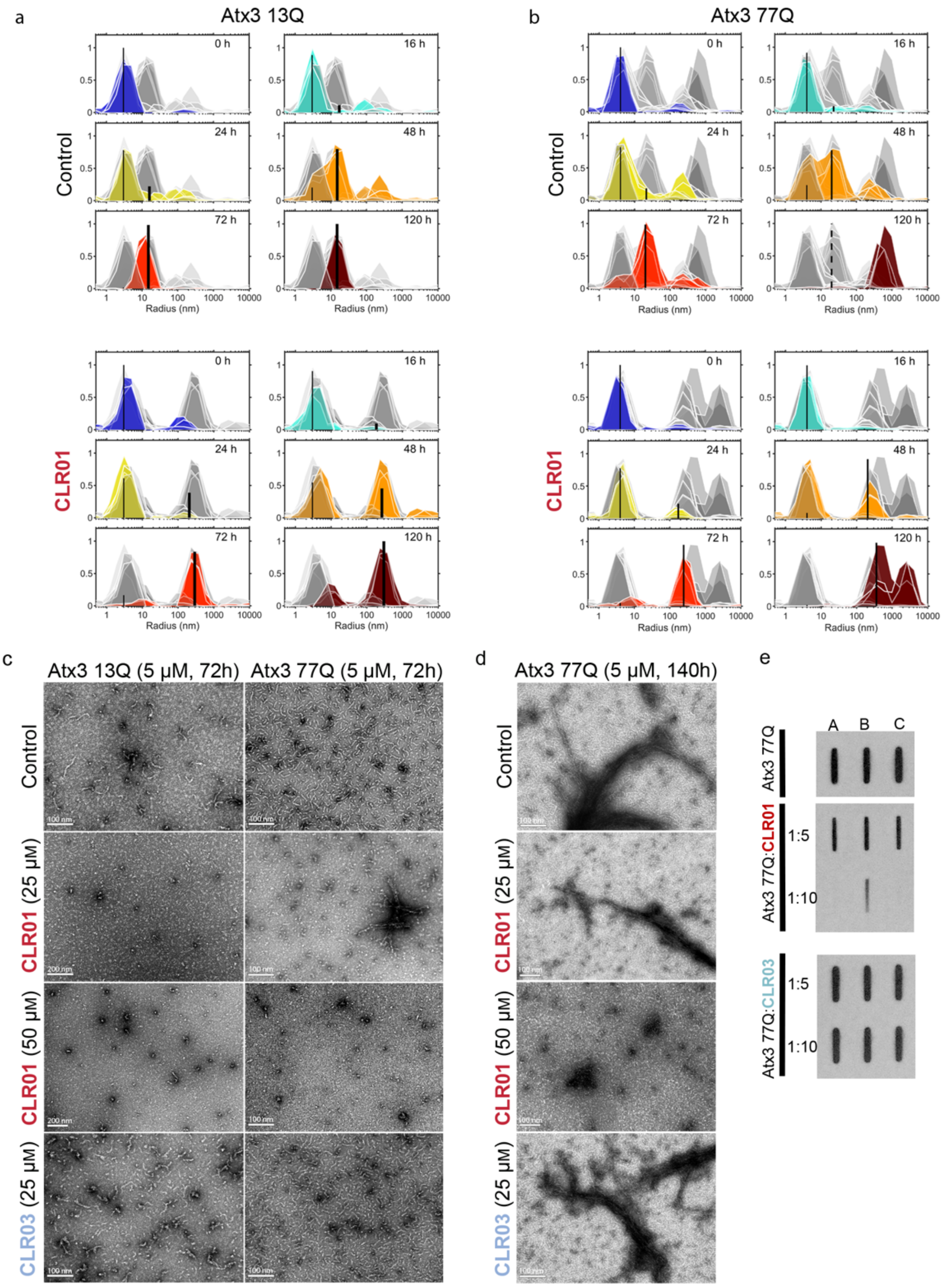

Figure S7 – CLR01 binding modifies the Atx3 aggregation pathway. Dynamic light scattering (DLS) intensity (a.u.) distribution at 0, 16, 24, 48, 72 or 120 h of aggregation of 5 µM Atx3 13Q (**a**) or Atx3 77Q (**b**) alone or in the presence of 25 µM CLR01 at 37 °C. Different colors show different time points (size distributions measured in triplicate). Each panel corresponds to a single time point, with the remaining time points represented in greyscale. The measured size distributions are represented separately for each time point, with the remaining time points represented in greyscale. Vertical lines: scattering intensities predicted by a nucleation-and-growth model for the population of monomers (thinner lines) and the population of amyloid-like fibrils (thicker lines); solid or dashed lines show good or bad agreements between model predictions and measured data. **c**) TEM analysis of the aggregate species formed by wild-type or mutant Atx3 after 72 h of incubation at 37 °C, in the absence or presence of CLR01 or CLR03. In the presence of 25 µM CLR01, a minor population of fibrillar agglomerates

were observed for Atx3 77Q. **d**) TEM analysis of the aggregate species formed at the endpoint of the aggregation assay of Atx3 77Q after 72 h of incubation at 37 °C in the absence or presence of CLR01 or CLR03. Scale bars correspond to 200 nm or 100 nm, as indicated in the images. **e**) Detection of mature SDS-resistant fibrils at the endpoint (t=66h) of 5 μM Atx3 77Q aggregation in the presence of 5- or 10-fold molar excess of CLR01 or CLR03 by filter retardation followed by immunoblot using anti-Atx3 1H9 antibody. A, B, and C are sample replicates.

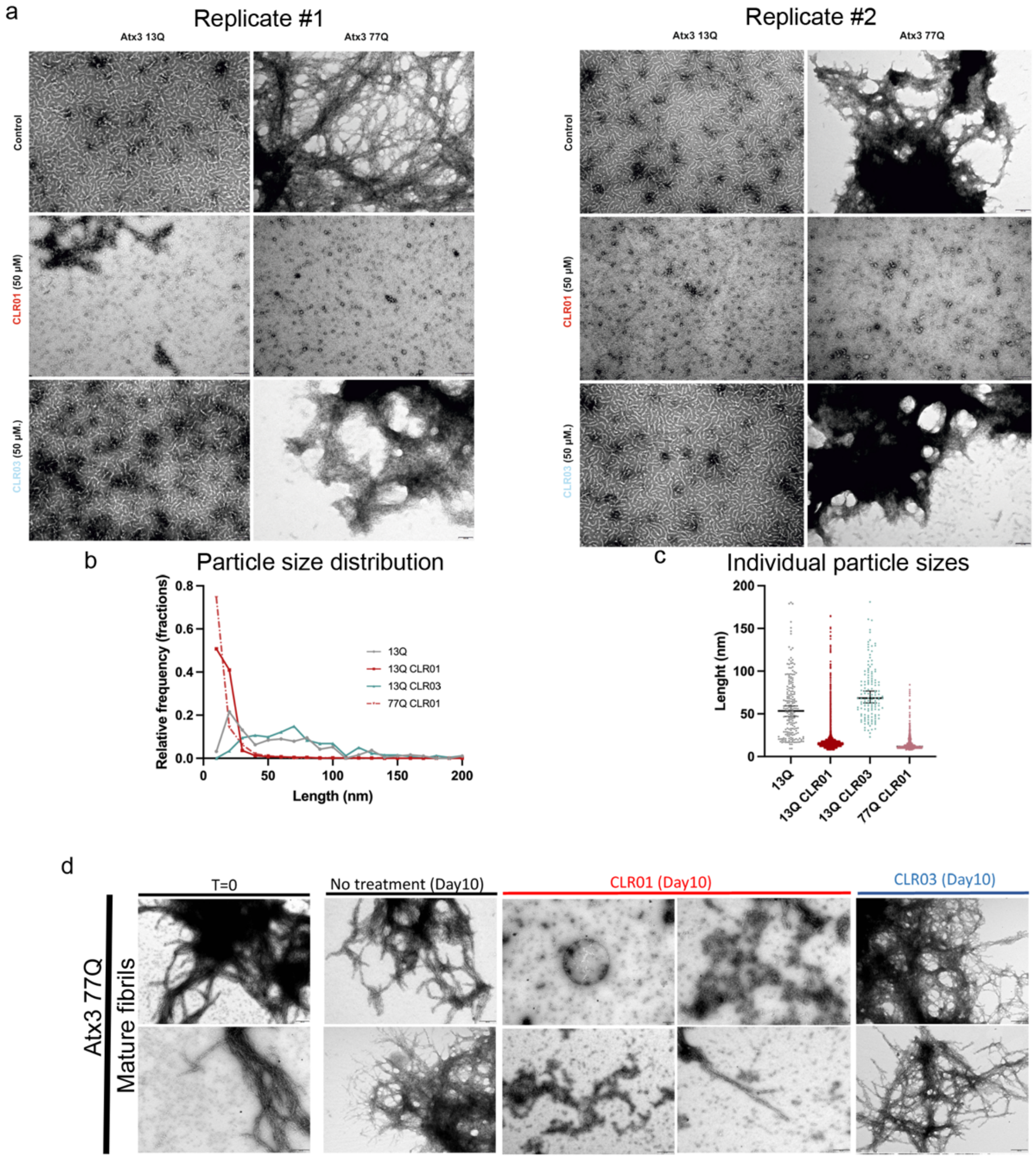

Figure S8 – Replicate Atx3 assembly and disassembly assays in the presence of CLR01 (1:10) and CLR03 (1:10). a) Representative TEM images for two independent replicates of Atx3 13Q and Atx3 77Q after ~140 hours at 37°C. Scale bars correspond to 100 nm. **b)** Particle size histograms, and **c)** scatter plots of all particle measurements (median ± 95% CI) from the two replicates shown in a) (two independent replicates, 4-10 images per condition). CLR01 shifts the particle population toward smaller, spherical Atx3 13Q oligomers compared with the control or with CLR03 incubation. Incubation of Atx3 77Q with CLR01 confirms this trend. Particle sizes were not determined for Atx77Q or Atx3 77Q:CLR03, due to the large fibrillar structures formed at extended incubation times. Descriptive statistics are provided in **Table S3 (Supporting information)**. **d)** Representative images of the morphological analysis of Atx3 77Q mature fibrils obtained at 160 h of incubation at 37 °C, before (T=0) and after 10 days of incubation in the absence (Control) or presence of 400 µM CLR01 or CLR03. Scale bars correspond to 100 nm.

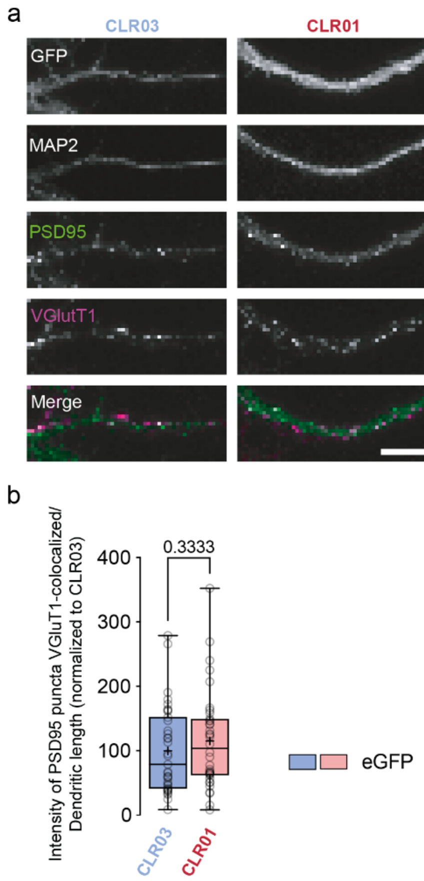

Figure S9 - CLR01 does not affect glutamatergic synaptic clusters in cortical cells. a) Immunolabelling of MAP2, PSD95 and VGluT1 in cortical neurons transfected with a plasmid encoding eGFP and incubated with CLR03 or CLR01. **b**) Fluorescence intensity of PSD95/vGLUT1 clusters was quantified (N=3 independent preparations per experiment, n=35-36 neurons per condition). Statistical analyses were performed using a t-test. Boxes show 25th and 75th percentiles, whiskers range from the minimum to the maximum values, and the horizontal line shows the median value. Mean is represented by "+".

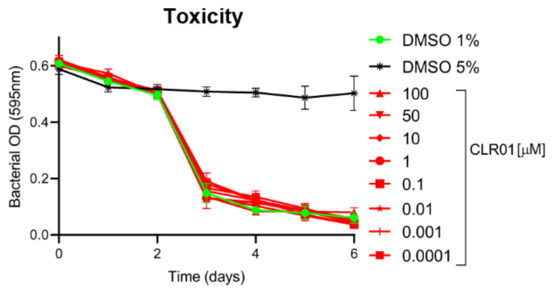

Figure S10 - Treatment of WT *C. elegans* with CLR01 compound did not affect animals' survival, development and fecundity. Graphs represent daily bacterial consumption measured by optical density (OD) at 595 nm of animals treated with CLR01, DMSO 1% (negative control) and 5% (positive control) were used as non-toxic and toxic treatment controls, respectively. For each condition tested, the mean OD was calculated for each day from three replicates and plotted over time. The highest safe concentration defined for CLR01 compound was of 100 µM, as the OD decrease paralleled the drug vehicle control samples. Raw data were statistically compared using a non-linear regression (curve fit) for sigmoidal curves and analyzed using a least squares model with 2 parameters (IC_{50} and Hill Slope values).

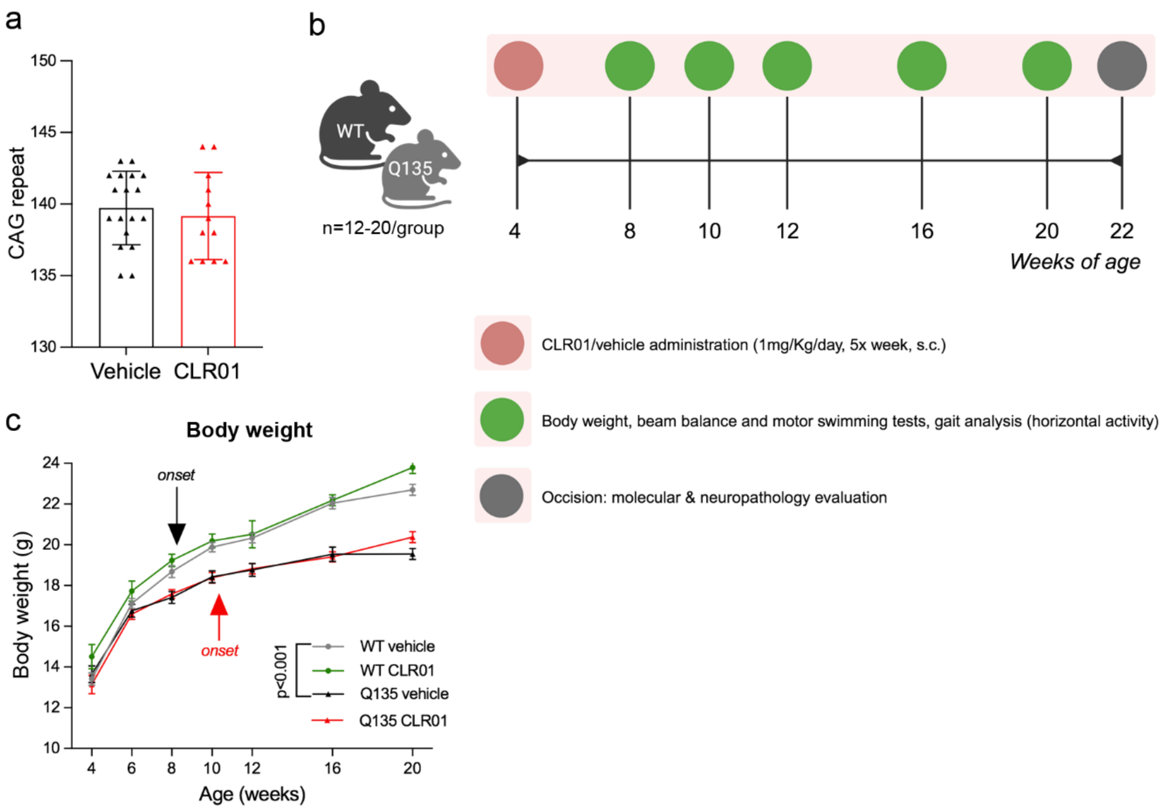

Figure S11 - Detailed description of the experimental groups used in the pre-clinical trial using CLR01. a) The CAG repeat length distribution was not different between vehicle- and CLR01-treated mice. **b**) Graphical representation of experiment timeline and behavioral testing. **c**) CLR01 treatment did not affect animals' body weight, both in SCA3 and WT mice. For (a) a Mann-Whitney U test was applied(N=13-19 SCA3 mice/group). For (c)arepeated measures ANOVA test (for group, genotype and treatment factors) was applied, followed by Tukey test for multiple comparisons. A one-way ANOVA test was applied, followed by a Tukey test for multiple comparisonsfor onset determination.Black arrowrepresents the comparisons between WT-vehicle and SCA3-vehicle. Red arrowrepresents the comparisons between SCA3-vehicle and SCA3-CLR01.(a,c)Data are represented as the mean±SEM. Detailed statistic report can be found in table S11.

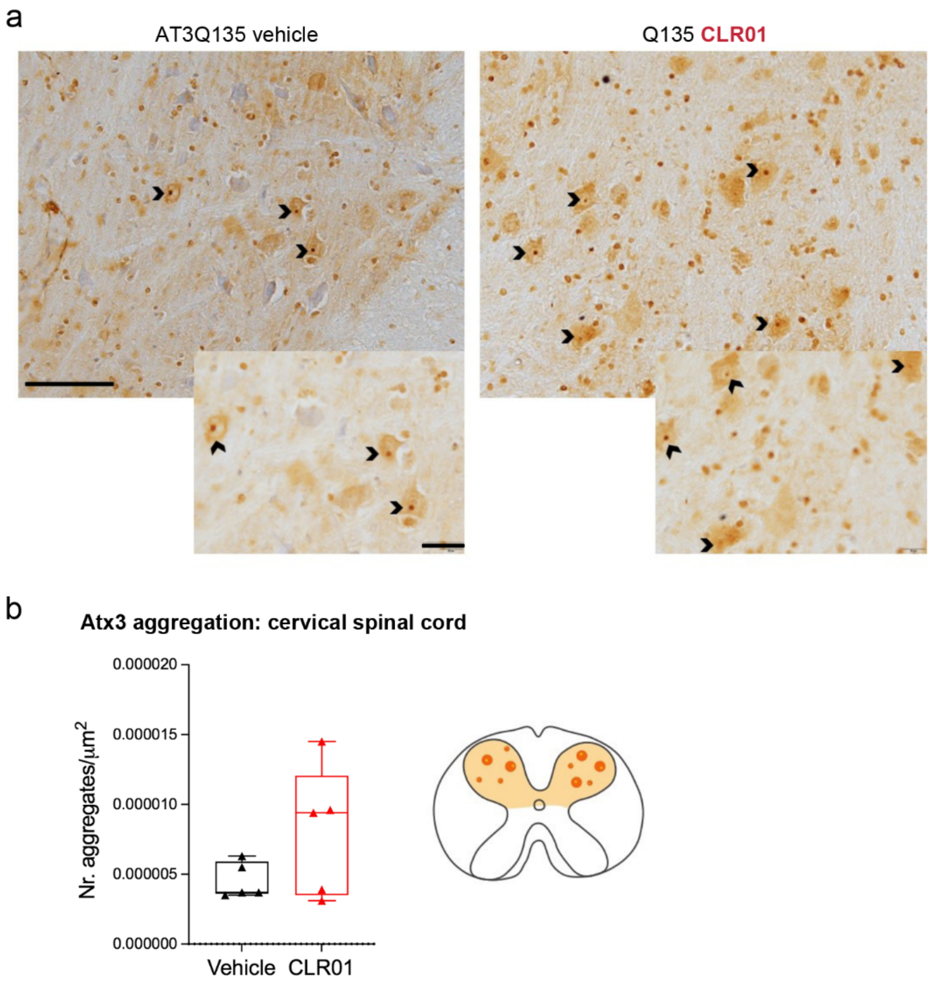

Figure S12 - Effect of CLR01 chronic administration on Atx3 aggregation in the cervical spinal cord of SCA3 mice. a) Representative images for Atx3 immunohistochemistry reveal intranuclear inclusions in the cervical spinal cord. **b)** Quantification of these end-stage nuclear Atx3-positive aggregates showed no statistically significant differences upon treatment with CLR01; nevertheless, a trend towards an increase by CLR01 treatment is supported by the large effect size observed (Cohen'd $d = 0.832$). Scale bars represent 100 µm for the lower magnification images and 20 µm for the higher magnification images. Sample size and statistical details are shown in **Supplementary Information Table S11**.

4. SUPPORTING TABLES

Table S1. CLR01 K128 binding site. Estimated fraction of contacts (%) between the JD sequence and CLR01 molecule at a 5Å distance over the GaMD trajectories (three replicas taken together). The results for the simulations of the JD in the open conformation in complex with nine CLR01 molecules on selected R/K residues (with a focus on the CLR01 complexed at position K128) and for the system containing a single CLR01 molecule at K128 are reported. Amino acids that displayed chemical shifts in the NMR HSQC spectra upon CLR01 binding to the JD are highlighted in red.

JD(open) + 9CLR01 simulations (contacts shown for CLR01 on K128)		JD(open) + CLR01 on K128 simulations	
Residue	Fraction	Residue	Fraction
M1	0.42%	M1	3.08%
**E2**	**0.03%**	**E2**	**0.25%**
I107	21.03%	P106	0.25%
**N108**	**30.28%**	I107	34.73%
E109	5.58%	**N108**	**38.60%**
R110	40.10%	E109	10.55%
**K125**	**87.18%**	R110	33.63%
L126	0.85%	R124	0.03%
**G127**	**83.95%**	**K125**	**78.30%**
**K128***	**100.00%**	L126	0.47%
Q129	19.35%	**G127**	**80.48%**
**W130**	**0.45%**	**K128***	**100.00%**
F131	0.17%	Q129	16.00%
L142	0.45%	**W130**	**0.80%**
D145	28.52%	F131	0.02%
T146	0.05%	L142	0.03%
D168	0.63%	S144	0.13%
D171	6.85%	D145	15.68%
C172	1.27%	T146	0.02%
**E173**	**9.28%**	D168	0.13%
**A174**	**3.37%**	D171	1.00%
**D175**	**8.63%**	C172	0.15%
**Q176**	**1.73%**	**E173**	**0.70%**
L177	2.25%	**A174**	**2.80%**
L178	21.93%	**D175**	**50.78%**
Q179	78.77%	**Q176**	**2.30%**
M180	56.58%	L177	30.33%
**I181**	**33.17%**	L178	39.48%
R182	43.52%	Q179	24.00%
		M180	27.10%
		**I181**	**36.27%**
		R182	17.88%

(*): Center of the binding site.

Table S2 – Report of statistical analysis of Atx3 13Q and Atx3 77Q ThT amyloid formation parameters (t_{50} and v_{50}) upon incubation with CLR01 or CLR03. Comparisons between conditions were carried out using the linear mixed-effects model (LME) (Atx3 13Q) or RM one-way ANOVA (77Q), followed by Dunnet's multiple comparisons test.

Figure	Atx3 variant	Median V_{50} ± SEM (h^{-1})	95% CI (h^{-1})	Statistical report	Sample size (n)
Fig. 2b (v_{50})	Atx3 13Q	0.1854 ± 0.03924	[0.07642, 0.2943]		5
	Atx3 13Q: CLR01	0.06426± 0.01316	[0.02774, 0.1008]	Atx3 13Q vs. Atx3 13Q: CLR01 (1:5)(p=0.0029)	5
	Atx3 13Q: CLR01	0.04679± 0.00999	[0.01903, 0.07455]	Atx3 13Q vs. Atx3 13Q: CLR01 (1:10)(p=0.0011)	5
	Atx3 13Q: CLR03	0.1533±0.05138	[-0.01020, 0.31680]	Atx3 13Q vs. Atx3 13Q: CLR03 (1:10)(p=0.7179)	4

	Atx3 variant	Median t_{50} ± SEM (h)	95% CI (h)	Statistical report	Sample size (n)
Fig. 2c (t_{50})	Atx3 13Q	15.79 ± 2.89	[7.77, 23,80]		5
	Atx3 13Q: CLR01 (1:5)	35.26± 12.54	[0,45, 70.07]	Atx3 13Q vs. Atx3 13Q: CLR01 (1:5)(p=0.1545)	5
	Atx3 13Q: CLR01 (1:10)	37.15± 13.85	[-1.29, 75.60]	Atx3 13Q vs. Atx3 13Q: CLR01 (1:10)(p=0.1116)	5
	Atx3 13Q: CLR03 (1:10)	18.36±4.08	[5,38, 31,35]	Atx3 13Q vs. Atx3 13Q: CLR03 (1:10)(p=0.9994)	4

	Atx3 variant	Median V_{50} ± SEM (h^{-1})	95% CI (h^{-1})	Statistical report	Sample size (n)
Fig. 2e (v_{50})	Atx3 77Q	0.1427 ± 0.02428	[0.06548, 0.2200]		4
	Atx3 77Q: CLR01 (1:5)	0.0536± 0.0155	[0.004293, 0.1030]	Atx3 13Q vs. Atx3 13Q: CLR01	4
	Atx3 77Q: CLR01	0.0401± 0.0117	[0.002950, 0.07736]	Atx3 13Q vs. Atx3 13Q: CLR01	4
	Atx3 77Q: CLR03	0.1533±0.0359	[0.02377, 0.2525]	Atx3 13Q vs. Atx3 13Q: CLR03	4

	Atx3 variant	Median t_{50} ± SEM (h)	95% CI (h)	Statistical report	Sample size (n)
Fig. 2f (t_{50})	Atx3 77Q	16.4 ± 2.77	[7.58, 25.23]		4
	Atx3 77Q: CLR01 (1:5)	33.48± 9.41	[3.54, 63.42]	Atx3 13Q vs. Atx3 13Q: CLR01 (1:5)(p=0.2828)	4
	Atx3 77Q: CLR01	43.48± 16.38	[-8.66, 95.61]	Atx3 13Q vs. Atx3 13Q: CLR01 (1:10)(p=0.0644)	4

	Atx3 77Q: CLR03	19.63±3.67	[7.96, 31,30]	Atx3 13Q vs. Atx3 13Q: CLR03 (1:10)(p=0.9778)	4

Table S3 – Summary of descriptive statistics for histogram data and individual particle size distributions derived from TEM image analysis.

**Figure**	**Atx3 variant**	**Median particle size (nm)**	**95% CI (nm)**	**Mean/ Std. Deviatio (SD)/Std. Error of Mean (SEM)**	**Sample size (n)**
Fig. 3b,c (Atx3 13Q and Atx3 77Q)	Atx3 13Q	33.18	[26.10, 43.58]	36.37/ 15.28/0.45	1166 (4 images)
	Atx3 13Q: CLR01	13.26	[10.60, 25.70]	19.02/11.62/0.49	559 (4 images)
	Atx3 13Q: CLR03	39.25	[32.68, 48.46]	39.71/18.41/0.55	931 (4 images)
	Atx3 77Q	38.85	[26.14, 52.80	43.79/23.50/1.02	526 (3 images)
	Atx3 77Q: CLR01	12.56	[10.40, 29.96]	22.21/18.92/0.54	559 (6 images)
	Atx3 77Q: CLR03	37.55	[26.38, 51.40]	42.52/24.42/1.08	511 (4 images)

**Figure**	**Atx3 variant**	**Median particle size (nm)**	**95% CI (nm)**	**Mean/ Std. Deviatio (SD)/Std. Error of Mean (SEM)**	**Sample size (n)**
Fig. S8b,c (Atx3 13Q and Atx3 77Q)	Atx3 13Q	53.21	[25.42, 80.20]	60.57/ 43.83/2.25	380 (4 images)
	Atx3 13Q: CLR01	14.92	[12.66, 18.03]	17.92/15.52/0.17	8150 (17 images)
	Atx3 13Q: CLR03	66.95	[46.67, 91.66]	78.68/52.43/3.23	263 (5 images)
	Atx3 77Q: CLR01	11.65	[10.48, 15.27]	15.80/12.43/0.40	973(10 images)

Table S4 - Report of statistical analysis performed in primary cortical neurons expressing the expanded (eGFP-Atx3 84Q) and non-expanded (eGFP-Atx3 28Q) forms of ataxin 3. Comparisons between conditions were carried out using Kruskal-Wallis Test followed by Dunn's multiple comparisons test.

Figure	Treatment	Transfection	Mean percentage of PSD95/vGluT1 puncta intensity ± SD	Statistical report	Sample size (n)
Fig. 4a-b. (Puncta intensity as a % of control)	N/A	eGFP	100 ± 78.37		3 (33cells)
		eGFP-Atx3 28Q	105.1 ± 83.63	eGFP vs. eGFP-atx3 28Q (	3 (36 cells)
		eGFP-Atx3 84Q	63.60 ± 53.88	eGFP-atx3 28Q vs. eGFP-atx3 84Q (p=0.0498)<br>eGFP-atx3 vs. eGFP-atx3 84Q (p=0.0912)	3 (36 cells)

Table S5 - Report of statistical analysis performed in primary cortical neurons treated with CLR01 and CLR03. Comparisons between conditions for each compound were carried out using Kruskal-Wallis Test followed by Dunn's multiple comparisons test.

Figure	Treatment	Transfection	Mean percentage of PSD95/vGluT1 puncta intensity ± SD	Statistical report	Sample size (n)
Fig. 4c-d. (Puncta intensity as a % of control)	CLR03	eGFP	100 ± 67.8		3 (35cell
		eGFP-Atx3 28Q	75.9 ± 77.3	eGFP vs. eGFP-atx3 28Q (p=0.612)	3 (41 cells)
		eGFP-Atx3 84Q	58.9 ± 58.28	eGFP vs. eGFP-atx3 84Q (p=0.0048<br>eGFP 28Q vs. eGFP-atx3 84Q (p>0.99	3 (40 cells)
	CLR01	eGFP	100 ± 63.2		3 (36 cells)
		eGFP-Atx3 28Q	97.1 ± 66.7	eGFP vs. eGFP-atx3 28Q (p>0.9999	3 (39 cells)
		eGFP-Atx3 84Q	72.7 ± 44.1	eGFP vs. eGFP-atx3 84Q (p=0.2081<br>eGFP 28Q vs. eGFP-atx3 84Q (p=0.38	3 (36 cells)

Table S6 - Statistical report of statistical analysis performed in primary cortical neurons treated with CLR01 and CLR03. Comparisons between each compound were carried out using Two-way ANOVA followed by Sidak's test for multiple comparisons.

Figure	Treatment	Transfection	Mean percentage of neurons with aggregate ± SD	Statistical report	Sample size (n)
Fig. 4e,f. (% of cell with aggregates)	CLR03	eGFP	0 ± 0	Interaction: $F_{(2,12)} =$ 0.1227, p = 0.8856, $\eta^2_p = 0.2708$ Treatment: $F_{(1,12)} =$ 0.06825, p = 0.7983, $\eta^2_p = 0.0753$ Transfection: $F_{(2,12)} =$ 39.15, p < 0.0001, $\eta^2_p = 83.41$	3 (31cells)
		eGFP-Atx3 28Q	31.2 ± 13.7		3 (32 cells)
		eGFP-Atx3 84Q	54.9 ± 5.0		3 (33 cells)
	CLR01	eGFP	0 ± 0		3 (32 cells)
		eGFP-Atx3 28Q	30.3 ± 22.9		3 (33 cells)
		eGFP-Atx3 84Q	59.9 ± 4.8		3 (35 cells)

Table S7 - Report of statistical analysis performed in primary cortical neurons treated with CLR01 and CLR03. Comparisons between each compound were carried out using Mann-Whitney test.

Figure	Treatment	Transfection	Mean percentage of PSD95/vGluT1 puncta intensity ± SD	Statistical report	Sample size (n)
Fig. S9. (Puncta intensity as a % of control)	CLR03	eGFP	100 ± 67.7	p=0.333	3 (35cells)
	CLR01	eGFP	115.1 ± 75.3		3 (36 cells)

Table S8 - Descriptive statistics regarding locomotion defects of AT3q130 animals upon chronic pre-symptomatic treatment with CLR01. Means of the locomotion defective phenotype are reported for all tested concentrations of CLR01. Comparisons between each concentration was carried out using One-way ANOVA followed by a bilateral Dunnett test for multiple comparisons. Efficiency was assessed using the following formula: Efficiency (%) $=\frac{\text{AT3q130}_{\text{DMSO}} - \text{AT3q130}_{\text{drug}}}{\text{AT3q130}_{\text{DMSO}} - \text{WT}}$.

Effect size was given by Cohen's d $=\frac{\text{Mean}_{\text{AT3q130}} - \text{Mean}_{\text{drug}}}{\text{Pooled SD}}$.

Strain	Concentration (µM)	Mean locomotion defective ± SD	Group comparison (Mean locomotion defective ± SD)	*P*-value	Effect Size	Efficiency (%)
**AT3q130 (AM685)**	0 (vehicle)	47.0 ± 8.7	WT 9.7 ± 4.5	***p*<0.001**		
	0.0001	25.9 ± 10.7	AT3q130 vehicle 46.8 ± 10.1	***p*=0.008**	2.85	56
	0.001	27.2 ± 9.2	AT3q130 vehicle 46.8 ± 10.1	***p*=0.014**	2.90	53
	0.01	22.4 ± 5.0	AT3q130 vehicle 46.8 ± 10.1	***p*=0.002**	4.36	66
	0.1	22.1 ± 7.0	AT3q130 vehicle 46.8 ± 10.1	***p*=0.001**	4.06	67
	1	28.6 ± 7.4	AT3q130 vehicle 46.8 ± 10.1	***p*=0.024**	2.95	50
	10	32.2 ± 8.9	AT3q130 vehicle 46.8 ± 10.1	*p*=0.100	2.20	40
	50	30.4 ± 12.4	AT3q130 vehicle 46.8 ± 10.1	*p*=0.052	2.08	45
	100	32.5 ± 7.9	AT3q130 vehicle 46.8 ± 10.1	*p*=0.112	2.26	39
**WT (N2)**	AT3q130 0.0001	25.9 ± 10.7	WT 9.7 ± 4.5		*p*=0.065	
	AT3q130 0.001	27.2 ± 9.2	WT 9.7 ± 4.5		*p*=0.040	
	AT3q130 0.01	22.4 ± 5.0	WT 9.7 ± 4.5		***p*=0.216**	
	AT3q130 0.1	22.1 ± 7.0	WT 9.7 ± 4.5		***p*=0.193**	
	AT3q130 1	28.6 ± 7.4	WT 9.7 ± 4.5		*p*=0.024	
	AT3q130 10	32.2 ± 8.9	WT 9.7 ± 4.5		*p*=0.005	
	AT3q130 50	30.4 ± 12.4	WT 9.7 ± 4.5		*p*=0.011	
	AT3q130 100	32.5 ± 7.9	WT 9.7 ± 4.5		*p*=0.004	

Table S9 – Descriptive statistics regarding the motility defects of AT3q130 animals upon post-symptomatic CLR01 treatment. Comparisons between groups was carried out using an independent-sample t-test.

Strain	Treatment	Concentration (μM)	Mean locomotion defective ± SD	Group comparison (Mean locomotion defective ± SD)	p-value
**AT3q130 (AM685)**	Day 4 (Before treatment)	0 (vehicle)	45.1 ± 2.3	WT 14.2 ± 1.3	***p*<0.001**
	Day 6	0 (vehicle)	53.9 ± 1.3	WT 24.4 ± 4.5	***p*<0.001**
		0.1	44.4 ± 3.9	AT3q130 vehicle 53.9 ± 1.3	***p*=0.004**
		0.1	44.4 ± 3.9	WT 24.4 ± 4.5	***p*=0.001**
	Day 8	0 (vehicle)	55.3 ± 9.1	WT 33.4 ± 6.4	***p*=0.008**
		0.1	41.0 ± 12.9	AT3q130 vehicle 55.3 ± 9.1	*p*=0.121
		0.1	41.0 ± 12.9	WT 33.4 ± 6.4	*p*=0.334

Table S10- Statistical report of CLR01 treatment in *C. elegans*. Effect size calculated using Uanhoro, J. O. (2017), available online at: https://effect-size-calculator.herokuapp.com/.

**Figure**		**Statistical report**	**Sample size (n)**
Fig. 5a (chronic treatment (motility))		F (8,29) = 3.500; p = 0.006; $\eta p^2 = 0.491$	4-5
Fig. 5b (WT chronic treatment (motility))		t (6) = 0.103; p = 0.921; g = 0.06	4
Fig. 5c (post-symptomatic treatment (motility))	Day 4	t (6) = 23.313; p < 0.001; g = 14.13	4
	Day 6	t (6) = 4.648; p = 0.004; g = 2.86	4
	Day 8	t (6) = 1.808; p = 0.121; g = 1.11	4
Fig. 5d	Number of aggregates/Total area	t (59) = 0.307; *p* = 0.760	31-30
	Area of aggregates	t (59) = 0.130; *p* = 0.897	31-30
Fig. 5e	Number of aggregates/Total area	t (60) = 0.883; *p* = 0.384	23-39
	Area of aggregates	t (58) = 1.235; *p* = 0.222	22-38
Fig. 5f	Number of aggregates/Total area	t (26) = 0.038; *p* = 0.970	16-12
	Area of aggregates	t (25) = 6.710; ***p* <0.001**	15-12
Fig. 5g	Number of aggregates/Total area	t (53) = 0.629; *p* = 0.532	27-28
	Area of aggregates	t (53) = 1.495; *p* = 0.141	27-28

Table S11. Reports of all statistical analyses performed in the mouse

Figure	Statistical report	Sample size
CAG repeat	U = 93.05, $p=0.536$	Q135 VEH, n=19<br>Q135 CLR01, n=13
Body weight	Group: $F_{(3, 33)}=12.94$, $p < 0.001$, $\eta^2_p = 0.546$<br>Genotype: $F_{(1, 33)}=36.67$, $p < 0.001$, $\eta^2_p = 0.512$<br>Treatment: $F_{(1, 33)}=0.270$, $p = 0.607$, $\eta^2_p = 0.008$	WT VEH, n=19<br>WT CLR01, n=14<br>Q135 VEH, n=19<br>Q135 CLR01, n=13
Motor swimming test	Group: $F_{(3, 48)}=23.36$, $p < 0.001$, $\eta^2_p = 0.594$<br>Genotype: $F_{(1, 48)}=43.97$, $p < 0.001$, $\eta^2_p = 0.478$<br>Treatment: $F_{(1, 48)}=7.72$, $p = 0.008$, $\eta^2_p = 0.138$	WT VEH, n=19<br>WT CLR01, n=14<br>Q135 VEH, n=19<br>Q135 CLR01, n=13
Beam Square 12mm	Group: $F_{(3, 45)}=16.43$, $p < 0.001$, $\eta^2_p = 0.523$<br>Genotype: $F_{(1, 45)}=44.74$, $p < 0.001$, $\eta^2_p = 0.499$<br>Treatment: $F_{(1, 45)}=3.16$, $p = 0.082$, $\eta^2_p = 0.066$	WT VEH, n=19<br>WT CLR01, n=14<br>Q135 VEH, n=19<br>Q135 CLR01, n=13
Gait quality	Group: $H_{(3)}=59.89$ $p<0.001$<br>Genotype: $H_{(1)}=48.46$; $p<0.001$<br>Treatment: $H_{(1)}=6.83$; $p=0.009$	WT VEH, n=19<br>WT CLR01, n=14<br>Q135 VEH, n=19<br>Q135 CLR01, n=13
Aggregation (Neuronal inclusions) in the SCC	$t_{(4,5)}= 6.748$, $p=0.165$; $d = 0.832$	5 mice/group (4-7 slices/mouse)
Neuropathology: Pyknotic cells in the SCC	Group: $F_{(2,14)}=16.011$, $p<0.001$; $\eta^2_p = 0.696$	WT VEH, n=6; Q135 VEH, n=6; Q135 CLR01, n=5(6 slices/mouse)
Neuropathology: Motor neurons in the SCC	Group: $F_{(3,16)}=10.397$, $p<0.001$; $\eta^2_p = 0.661$	WT VEH, n=6; WT CLR01, n=5; Q135 VEH, n=5; Q135 CLR01, n=5(4-6 slices/mouse)

5. REFERENCES

[1] Y. Miao, V. A. Feher, J. A. McCammon, *J Chem Theory Comput* **2015**, *11* (8), 3584, https://doi.org/10.1021/acs.jctc.5b00436.

[2] G. Nicastro, R. P. Menon, L. Masino, P. P. Knowles, N. Q. McDonald, A. Pastore, *Proc Natl Acad Sci U S A* **2005**, *102* (30), 10493, https://doi.org/10.1073/pnas.0501732102.

[3] Y. Mao, F. Senic-Matuglia, P. P. Di Fiore, S. Polo, M. E. Hodsdon, P. De Camilli, *Proc Natl Acad Sci U S A* **2005**, *102* (36), 12700, https://doi.org/10.1073/pnas.0506344102.

[4] S. Jo, T. Kim, V. G. Iyer, W. Im, *J Comput Chem* **2008**, *29* (11), 1859, https://doi.org/10.1002/jcc.20945.

[5] J. Huang, S. Rauscher, G. Nawrocki, T. Ran, M. Feig, B. L. de Groot, H. Grubmuller, A. D. MacKerell, Jr., *Nat Methods* **2017**, *14* (1), 71, https://doi.org/10.1038/nmeth.4067.

[6] J. Lee, X. Cheng, J. M. Swails, M. S. Yeom, P. K. Eastman, J. A. Lemkul, S. Wei, J. Buckner, J. C. Jeong, Y. Qi, S. Jo, V. S. Pande, D. A. Case, C. L. Brooks, 3rd, A. D. MacKerell, Jr., J. B. Klauda, W. Im, *J Chem Theory Comput* **2016**, *12* (1), 405, https://doi.org/10.1021/acs.jctc.5b00935.

[7] W. L. Jorgensen, J. Chandrasekhar, J. D. Madura, R. W. Impey, M. L. Klein, *J Chem Phys* **1983**, *79* (2), 926, https://doi.org/Doi 10.1063/1.445869.

[8] W. L. Jorgensen, C. Jenson, *J Comput Chem* **1998**, *19* (10), 1179, https://doi.org/Doi 10.1002/(Sici)1096-987x(19980730)19:10<1179::Aid-Jcc6>3.0.Co;2-J.

[9] K. Vanommeslaeghe, E. Hatcher, C. Acharya, S. Kundu, S. Zhong, J. Shim, E. Darian, O. Guvench, P. Lopes, I. Vorobyov, A. D. MacKerell, *J Comput Chem* **2010**, *31* (4), 671, https://doi.org/10.1002/jcc.21367.

[10] A. Meiners, S. Backer, I. Hadrovic, C. Heid, C. Beuck, Y. B. Ruiz-Blanco, J. Mieres-Perez, M. Porschke, J. N. Grad, C. Vallet, D. Hoffmann, P. Bayer, E. Sanchez-Garcia, T. Schrader, S. K. Knauer, *Nat Commun* **2021**, *12* (1), 1505, https://doi.org/10.1038/s41467-021-21753-9; N. Samanta, Y. B. Ruiz-Blanco, Z. Fetahaj, D. Gnutt, C. Lantz, J. A. Loo, E. Sanchez-Garcia, S. Ebbinghaus, *Chembiochem* **2022**, *23* (21), e202200396, https://doi.org/10.1002/cbic.202200396.

[11] T. Darden, D. York, L. Pedersen, *J Chem Phys* **1993**, *98* (12), 10089, https://doi.org/Doi 10.1063/1.464397.

[12] D. A. Case, K. Belfon, I. Y. Ben-Shalom, S. R. Brozell, D. S. Cerutti, T. E. Cheatham, V. W. D. C. III, T. A. Darden, R. E. Duke, G. Giambasu, M. K. Gilson, H. Gohlke, R. H. A.W. Goetz, S. Izadi, S. A. Izmailov, K. Kasavajhala, A. Kovalenko, R. Krasny, T. Kurtzman, T. S. Lee, S. LeGrand, P. Li, C. Lin, J. Liu, T. Luchko, R. Luo, V. Man, K. M. Merz, Y. Miao, O. Mikhailovskii, G. Monard, H. Nguyen, A. Onufriev, F. Pan, S. Pantano, R. Qi, D. R. Roe, A. Roitberg, C. Sagui, S. Schott-Verdugo, J. Shen, C. L. Simmerling, N. R. Skrynnikov, J. Smith, J. Swails, R. C. Walker, J. Wang, L. Wilson, R. M. Wolf, X. Wu, Y. Xiong, Y. Xue, D. M. York, P. A. Kollman, in The University of California, San Francisco **2020**.

[13] W. Humphrey, A. Dalke, K. Schulten, *J Mol Graph* **1996**, *14* (1), 33, https://doi.org/10.1016/0263-7855(96)00018-5.

[14] W. L. DeLano, The PyMOL molecular graphics system. http://www.pymol.org. **2002**.

[15] Y. B. Ruiz-Blanco, E. Sanchez-Garcia, *J Chem Theory Comput* **2020**, *16* (3), 1396, https://doi.org/10.1021/acs.jctc.9b00725.

[16] J. C. Phillips, D. J. Hardy, J. D. C. Maia, J. E. Stone, J. V. Ribeiro, R. C. Bernardi, R. Buch, G. Fiorin, J. Hénin, W. Jiang, R. McGreevy, M. C. R. Melo, B. K. Radak, R. D. Skeel, A. Singharoy, Y. Wang, B. Roux, A. Aksimentiev, Z. Luthey-Schulten, L. V. Kale, K. Schulten, C. Chipot, E. Tajkhorshid, *J Chem Phys* **2020**, *153* (4), https://doi.org/Artn 044130 10.1063/5.0014475.

[17] A. Silva, B. Almeida, J. S. Fraga, P. Taboada, P. M. Martins, S. Macedo-Ribeiro, *Angew Chem Int Ed Engl* **2017**, *56* (45), 14042, https://doi.org/10.1002/anie.201707345.
[18] Z. Sarkany, F. Rocha, A. Bratek-Skicki, P. Tompa, S. Macedo-Ribeiro, P. M. Martins, *Adv Sci (Weinh)* **2023**, *10* (23), e2301501, https://doi.org/10.1002/advs.202301501.
[19] J. Schäfer, MatScat (https://www.mathworks.com/matlabcentral/fileexchange/36831-matscat), accessed.
[20] G. Bitan, A. Lomakin, D. B. Teplow, *J Biol Chem* **2001**, *276* (37), 35176, https://doi.org/10.1074/jbc.M102223200; F. Rahimi, P. Maiti, G. Bitan, *J Vis Exp* **2009**, (23), https://doi.org/10.3791/1071.
[21] F. Figueiredo, M. Lopes-Marques, B. Almeida, N. Matscheko, P. M. Martins, A. Silva, S. Macedo-Ribeiro, *Cells* **2022**, *11* (12), https://doi.org/10.3390/cells11121969.
[22] P. Talbiersky, F. Bastkowski, F. G. Klärner, T. Schrader, *J. Am. Chem. Soc.* **2008**, *130* (30), 9824, https://doi.org/Doi 10.1021/Ja801441j; D. H. Lopes, A. Attar, G. Nair, E. Y. Hayden, Z. Du, K. McDaniel, S. Dutt, K. Bravo-Rodriguez, S. Mittal, F. G. Klarner, C. Wang, E. Sanchez-Garcia, T. Schrader, G. Bitan, *ACS Chem Biol* **2015**, *10* (6), 1555, https://doi.org/10.1021/acschembio.5b00146.
[23] H. Shahpasand-Kroner, I. Siddique, R. Malik, G. R. Linares, M. I. Ivanova, J. Ichida, T. Weil, J. Munch, E. Sanchez-Garcia, F. G. Klarner, T. Schrader, G. Bitan, *Pharmacol Rev* **2023**, *75* (2), 263, https://doi.org/10.1124/pharmrev.122.000654.
[24] G. Nicastro, M. Habeck, L. Masino, D. I. Svergun, A. Pastore, *J Biomol NMR* **2006**, *36* (4), 267, https://doi.org/10.1007/s10858-006-9092-z.
[25] E. Jurrus, D. Engel, K. Star, K. Monson, J. Brandi, L. E. Felberg, D. H. Brookes, L. Wilson, J. Chen, K. Liles, M. Chun, P. Li, D. W. Gohara, T. Dolinsky, R. Konecny, D. R. Koes, J. E. Nielsen, T. Head-Gordon, W. Geng, R. Krasny, G. W. Wei, M. J. Holst, J. A. McCammon, N. A. Baker, *Protein Sci* **2018**, *27* (1), 112, https://doi.org/10.1002/pro.3280.
